# Real-world evidence indicates romosozumab use is associated with a greater reduction in osteoporotic fractures than PTH (1–34) analogs in women

**DOI:** 10.1186/s13293-025-00817-1

**Published:** 2026-01-03

**Authors:** Ko-Hsiu Lu, Shiow-Ing Wang, Shun-Fa Yang

**Affiliations:** 1 Department of Orthopedics, Chung Shan Medical University Hospital, Taichung, Taiwan; 2 School of Medicine, Chung Shan Medical University, Taichung, Taiwan; 3 Center for Health Data Science, Department of Medical Research, Chung Shan Medical University Hospital, Taichung, Taiwan; 4 Department of Health Policy and Management, College of Health Care and Management, Chung Shan Medical University, Taichung, Taiwan; 5 Institute of Medicine, Chung Shan Medical University, Taichung, Taiwan; 6 Department of Medical Research, Chung Shan Medical University Hospital, Taichung, Taiwan

**Keywords:** Abaloparatide, Osteoporotic fracture, Romosozumab, Teriparatide, TriNetX

## Abstract

**Background:**

To compare the effectiveness of romosozumab (ROMO) with parathyroid hormone (PTH) receptor agonists [teriparatide (TPTD)/abaloparatide (APTD)] in reducing fracture risk following osteoporosis treatment.

**Methods:**

A TriNetX cohort study assessed fracture and mortality risks using Kaplan–Meier analysis with hazard ratios (HRs) and 95% confidence intervals (CIs).

**Results:**

After propensity score matching (n = 2,258 pairs), ROMO users had lower risks of osteoporotic fractures (HR: 0.711, 95% CI: 0.542–0.931) and hypercalcemia (HR = 0.707, 95% CI: 0.511–0.977) compared with PTH receptor agonists. Five subgroup analyses demonstrated a reduced fracture risk in the ROMO cohort among women (HR: 0.738), patients aged ≥ 65 years (HR: 0.652), individuals with a history of prior fractures (HR: 0.659), and those without chronic kidney disease (CKD) (HR: 0.731). Sensitivity analyses confirmed the robustness of the findings across different covariate adjustments, data sources, and extended follow-up, consistently showing a lower risk of hypercalcemia and nonsignificant trends toward reduced fracture risk in the ROMO cohort.

**Conclusion:**

Compared with PTH analogs, ROMO offers stronger short-term protection against osteoporotic fractures and hypercalcemia, particularly in older women with prior fractures. Nonetheless, cardiovascular safety, calcium metabolism, and sequential therapy require careful consideration for individualized treatment.

**Supplementary Information:**

The online version contains supplementary material available at 10.1186/s13293-025-00817-1.

## Plain language summary

Osteoporosis is a disease that makes bones weak and more likely to break, especially in older adults and women after menopause. This study compared two types of bone-strengthening medicines: romosozumab (ROMO) and parathyroid hormone (PTH) drugs, including teriparatide and abaloparatide. Researchers used a large health database to see which treatment better prevented bone fractures. They found that people taking ROMO had fewer fractures and fewer cases of high calcium levels in the blood than those taking PTH drugs. The benefits of ROMO were strongest in women, people aged 65 and older, and those who had fractures before. These results suggest that ROMO may offer stronger short-term protection against fractures compared with PTH drugs. However, doctors still need to consider possible side effects and each person’s overall health before choosing the best treatment. This study provides helpful information for patients and doctors in deciding how to manage osteoporosis and reduce the risk of fragility fractures.

**Supplementary Information:**

The online version contains supplementary material available at 10.1186/s13293-025-00817-1.

## Highlights

Compared romosozumab (ROMO) with parathyroid hormone (PTH) receptor agonists for fracture prevention in osteoporosis.

ROMO users had lower risks of fractures and hypercalcemia after patient matching.

Benefits were greater in women, adults ≥ 65 years, those with prior fractures, and without kidney disease.

Findings were consistent across data sources and sensitivity analyses.

ROMO offers stronger short-term protection, though safety and treatment sequencing need consideration.

**Supplementary Information:**

The online version contains supplementary material available at 10.1186/s13293-025-00817-1.

## Background

Severe osteoporosis with high fracture risk requires uninterrupted, long-term therapy [1]. Antiresorptive agents, including bisphosphonates (BPs), remain first-line due to proven efficacy, clinical experience, convenient dosing, and cost-effectiveness [1, 2]. However, osteoporotic fractures, particularly vertebral and hip fractures, substantially impact on morbidity, quality of life, mortality, and healthcare burdens, ultimately shortening life expectancy [3]. Patients with very high fracture risk, such as severe bone loss, prior vertebral fracture or hip fracture, antiresorptive-related adverse events, or poor bone health requiring surgery, may benefit from rapid gains in bone mass, microarchitecture, and biomechanics through osteoanabolic agents [4, 5]. These include teriparatide (TPTD, Forteo®), abaloparatide (APTD, Tymlos®), and romosozumab (ROMO, Evenity®), which provide greater bone mineral density (BMD) gains and fracture risk reduction than BPs [1, 6, 7].

TPTD, 1–34 parathyroid hormone (PTH) receptor 1 agonist, predominantly stimulates remodeling-based bone formation [1, 8]. Approved by the U.S. Food and Drug Administration (FDA) in 2002, TPTD was originally limited to a 24-month lifetime treatment duration [3, 9], but this restriction was removed in 2020 [10, 11]. Although it increases bone formation, coupled resorption constrains the net anabolic benefit [12]. In pivotal trials, 24 months of daily 20 µg TPTD reduced clinical fractures by 52%, vertebral fractures by 56%, and non-vertebral fractures by 34% compared with risedronate [13] with significant benefit seen as early as seven months [14]. Versus placebo, TPTD decreased vertebral fracture risk by 65% and non-vertebral fracture risk by 53% at 21 months [15].

APTD, a 1–34 PTH-related peptide analog and PTH receptor 1 agonist, was U.S. FDA-approved in 2017 with a 24-month lifetime limit. In an 18-month trial, daily 80 µg subcutaneous APTD increased BMD by 10.4% at the lumbar spine, 4.3% at the total hip, and 4.0% at the femoral neck versus placebo [16]. Both APTD and TPTD significantly reduced vertebral fracture risk by 86% and 80%, respectively. While APTD showed lower major osteoporotic fracture incidence than TPTD (1.5% vs. 3.1%), non-vertebral fracture reduction did not differ significantly (43% vs. 28%) from placebo.

ROMO, a humanized IgG2 monoclonal antibody against sclerostin, stimulates bone formation and inhibits resorption, primarily via modeling-based formation, producing rapid, substantial BMD gains [1, 8]. Approved by the U.S. FDA in 2019 for postmenopausal women, ROMO treatment is limited to 12 months [17]. Monthly 210 mg ROMO reduced clinical fractures by 27% versus alendronate, vertebral fractures by 48%, non-vertebral fractures by 19%, and hip fractures by 38%. Compared with placebo, ROMO reduced clinical fractures by 36% and vertebral fractures by 73%, with the strongest effect at the spine [18, 19]. After 12 months, ROMO increases femoral neck BMD by 4.8–5.9% versus placebo and 2.5–3.2% versus alendronate, total hip BMD by 4.8–6.8% versus placebo and 2.2–3.4% versus alendronate, and lumbar spine BMD by 11.4–13.3% versus placebo and 7.2–8.7% versus alendronate [8, 18, 19].

TPTD is often reserved for patients at very high fracture risk (e.g., ≥ 2 prevalent fractures) or prior BP exposure [4]. However, prior antiresorptive therapy may blunt TPTD’s anabolic response, particularly at the hip, sometimes causing early BMD decline in the first year [20, 21]. Similarly, early ROMO responses are attenuated by prior BPs or denosumab [21, 22], predictable by early changes in bone turnover markers [23, 24]. Head-to-head trials in treatment-naïve postmenopausal women with osteopenia show greater BMD and bone strength gains at the femoral neck, total hip, and lumbar spine after 12 months of ROMO versus TPTD, especially at the lumbar spine [21, 25, 26]. The STRUCTURE trial confirmed superior BMD improvements in women transitioning from BPs to ROMO versus TPTD [27].

Because on-treatment BMD gains strongly correlate with fracture risk reduction [28], attenuation after prior antiresorptive therapy may compromise both agents’ effectiveness. Given the lack of real-world data directly comparing fracture outcomes between ROMO and PTH receptor 1 agonists, we analyzed the TriNetX U.S. network in osteoporosis patients without recent fractures (within three months), evaluating osteoporotic, vertebral, non-vertebral, and hip fractures, all-cause mortality, and calcium abnormalities.

## Materials and methods

### Study design

We conducted a retrospective cohort study using the TriNetX U.S. collaborative network, which includes 72 healthcare organizations (HCOs) and over 100 million patients across the United States. This network captures both insured and uninsured patients from hospitals, as well as primary care and specialty services. Detailed information on TriNetX is available on its official website (https://trinetx.com/?mc_cid=7e2ecd5bc5&mc_eid=%5BUNIQID%5D). Data quality is ensured through standardized metrics assessing conformance, completeness, and plausibility. Using the TriNetX platform, we created matched comparison cohorts with a built-in propensity score matching (PSM) algorithm according to predefined inclusion and exclusion criteria [29, 30]. Analyses covered the period from January 1, 2019, to December 31, 2024.

### Ethics statement

The TriNetX database contains only de-identified data. Protected health information, including exact age for patients over 90 years, was removed. De-identification has been validated by a qualified expert in accordance with the HIPAA Privacy Rule (§164.514(b)(1)), most recently in December 2020. Therefore, analyses using TriNetX are generally not considered “human subjects research” and are exempt from IRB review. This study was additionally approved by the Institutional Review Board of Chung Shan Medical University Hospital (approval no. CS2-23,208) and was reported in accordance with the RECORD (Reporting of Studies Conducted Using Observational Routinely Collected Health Data) guidelines.

### Participants

**Fig. 1:**
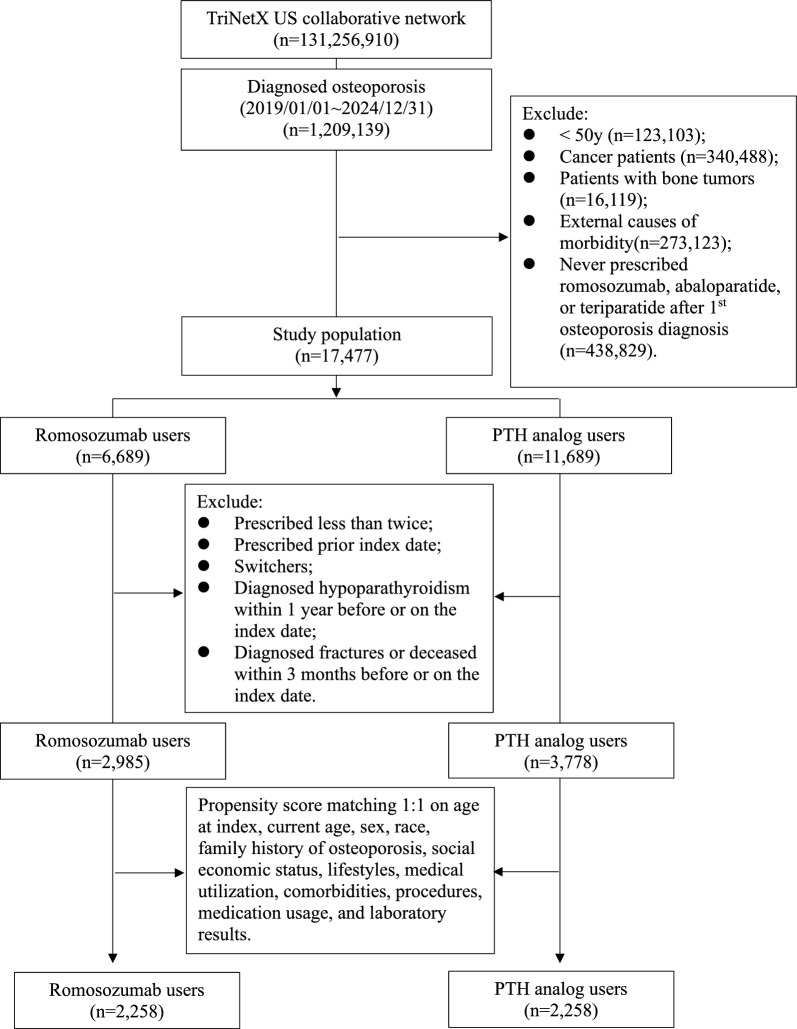
Flowchart of the selection process

Patients diagnosed with osteoporosis were identified on at least two separate occasions using the International Classification of Diseases, Tenth Revision, Clinical Modification (ICD-10-CM) codes M80 and M81. Eligible patients were aged ≥ 50 years and received either ROMO (defined by the normalized name for clinical drugs, RxNorm 2,123,126), TPTD (RxNorm 32,915), or APTD (RxNorm 1,921,069) following their initial recorded diagnosis of osteoporosis (Fig. 1).

The index date was defined as the first prescription date of ROMO, TPTD, or APTD during the study period. Exclusion criteria included: prevalent users who received prescriptions for ROMO, TPTD, or APTD before January 1, 2019, or prior to initial osteoporosis diagnosis; history of cancer (C00–C96), bone tumors (D16, D48.0, and D49.2), or external causes of morbidity (V00–Y99); prior exposure to comparator drugs before the index date; age < 50 years at the index date, fewer than two prescriptions of the study drugs, hypoparathyroidism (E20) within one year before the index date, or diagnosed fractures or deceased within three months before or on the index date; and patients who switched between ROMO and TPTD/APTD after the index date [e.g., patients in the ROMO cohort must not have received TPTD or APTD, and vice versa].

### Covariates

Baseline covariates associated with fracture risk, including demographics, comorbidities, and medication use, were assessed during the year preceding the index date. To minimize confounding, differences between ROMO users and the TPTD/APTD users were adjusted. Detailed covariates and their corresponding codes are provided in Supplementary Table 1.

### Outcomes

Patients were followed from one day after the index date to 12 months thereafter. The primary outcome was osteoporotic fractures, categorized as vertebral fractures and non-vertebral fractures. vertebral fractures included cervical vertebra and neck fractures (S12), thoracic vertebral fractures (S22.0), lumbar vertebral fractures (S32.0), fatigue fractures of vertebra (M48.4), and collapsed vertebra not classified elsewhere (M48.5). Non-vertebral fractures included pelvic fractures (ilium [S32.3], acetabulum [S32.4], pubis [S32.5], ischium [S32.6], other pelvic fractures [S32.8]), hip fractures (head/neck of femur [S72.0], pertrochanteric [S72.1]), other femur fractures (S72), lower leg fractures including ankle (S82), shoulder/upper arm fractures (S42), and forearm fractures (S52). Secondary outcomes included all-cause mortality, hypocalcemia (E83.51), and hypercalcemia (E83.52) as positive control outcomes**.**

### Subgroup analyses

We examined whether fracture, mortality, and calcium abnormality risks varied according to age, sex, prior fracture history, circulatory system disease, and chronic kidney disease (CKD). Age was stratified into 50–64 years and ≥ 65 years; sex as male or female; fracture history as presence or absence of fractures occurring three months to three years before the index date; and circulatory disease and CKD as present or absent.

### Sensitivity analysis

To assess the robustness of the results, four sensitivity analyses were performed: (1) propensity score matching was performed using an alternative set of covariates, (2) addressing geographic variation by using global collaborative network, which includes more than 120 HCOs across 19 countries (the United Kingdom, Italy, Spain, Germany, Poland, Bulgaria, Lithuania, Brazil, India, Australia, Singapore, Malaysia, Taiwan, Japan, and the United States), thereby enhancing external validity in a more diverse population, (3) modified control cohort restricted to TPTD users only, and (4) given that romosozumab is typically limited to 12 months of use according to regulatory labeling and clinical guidelines, whereas TPTD may be administered for up to 24 months, we conducted a sensitivity analysis to address potential bias arising from the different approved treatment durations.

### Statistical analysis

PSM was used to create cohorts with similar baseline characteristics, controlling for confounders. Matching was performed using a 1:1 greedy nearest-neighbor approach (TriNetX support documentation: https://support.trinetx.com/hc/en-us/articles/360011978033-In-compare-outcomes-how-are-patients-matched-when-balancing-cohorts/). Baseline balance was assessed using standardized mean differences (SMDs), with SMD < 0.1 considered well matched [31]. Kaplan–Meier survival analysis estimated outcome probabilities, with hazard ratios (HRs) and 95% confidence intervals (CIs) calculated using the *survival* package v.3.2–3 in R (R Foundation, Vienna, Austria), following TriNetX methodology (https://support.trinetx.com/hc/en-us/articles/360053133594-How-does-TriNetX-test-for-proportionality-on-a-hazard-ratio-). Log-rank tests determined differences between survival curves. Two-tailed p-values < 0.05 were considered statistically significant, and the proportional hazards assumption was verified using scaled Schoenfeld residuals.

## Results

### Baseline characteristics

A total of 17,477 patients were included in the present study. After applying propensity score matching, 2,258 users of ROMO and an equal number of PTH analog users were selected for analysis. The selection process is illustrated in Fig. 1. Table 1 summarizes the demographics, comorbidities, procedures, medications, and laboratory data of ROMO and PTH analog users before and after propensity score matching. Compared with TPTD/APTD users, ROMO users were generally older and more likely to be female at baseline. Additionally, fewer patients with cardiovascular disease were identified in the ROMO group. Most baseline characteristics were well balanced between the two groups (all standardized mean differences [SMDs] < 0.1), except for the use of antidepressants, antiepileptics, and alendronate.

**Table 1:** Baseline characteristics of study subjects before and after PSM matching (PSM)

Variables	Before PSM^a^			After PSM^a^		
	Romosozumab users (n = 2985)	PTH analogs users (n = 3778)	SMD	Romosozumab users (n = 2258)	PTH analogs users (n = 2258)	SMD
Current age, mean ± SD	70.9 ± 7.5	69.3 ± 7.7	0.207	70.1 ± 7.4	70.1 ± 7.7	0.007
Age at index, mean ± SD	68.7 ± 7.5	66.2 ± 7.7	0.326	67.6 ± 7.3	67.6 ± 7.6	0.002
Sex, n (%)						
Female	2867 (96.0)	3381 (89.5)	0.255	2173 (96.2)	2169 (96.1)	0.009
Male	30 (1.0)	324 (8.6)	0.360	30 (1.3)	33 (1.5)	0.011
Unknown gender	88 (2.9)	73 (1.9)	0.066	55 (2.4)	56 (2.5)	0.003
Race, n (%)						
White	2339 (78.4)	3020 (79.9)	0.039	1809 (80.1)	1826 (80.9)	0.019
Unknown race	211 (7.1)	346 (9.2)	0.077	173 (7.7)	168 (7.4)	0.008
Asian	292 (9.8)	169 (4.5)	0.207	154 (6.8)	144 (6.4)	0.018
Other race	60 (2.0)	137 (3.6)	0.098	58 (2.6)	60 (2.7)	0.006
Black or African American	63 (2.1)	90 (2.4)	0.018	54 (2.4)	51 (2.3)	0.009
Native Hawaiian or other Pacific islander	13 (0.4)	10 (0.3)	0.029	10 (0.4)	10 (0.4)	0.000
American Indian or Alaska native	10 (0.3)	11 (0.3)	0.008	10 (0.4)	10 (0.4)	0.000
Family history of osteoporosis, n (%)	46 (1.5)	53 (1.4)	0.011	42 (1.9)	39 (1.7)	0.010
Social economic status, n (%)						
Persons with potential health hazards related to socioeconomic and psychosocial circumstances	14 (0.5)	12 (0.3)	0.024	10 (0.4)	10 (0.4)	0.000
Lifestyles, n (%)						
Personal history of nicotine dependence	140 (4.7)	200 (5.3)	0.028	108 (4.8)	115 (5.1)	0.014
Nicotine dependence	62 (2.1)	135 (3.6)	0.090	53 (2.3)	53 (2.3)	0.000
Tobacco use	25 (0.8)	39 (1.0)	0.020	19 (0.8)	24 (1.1)	0.023
Alcohol related disorders	12 (0.4)	17 (0.5)	0.007	10 (0.4)	10 (0.4)	0.000
Reduced mobility	18 (0.6)	20 (0.5)	0.010	11 (0.5)	13 (0.6)	0.012
Difficulty in walking, not elsewhere classified	12 (0.4)	10 (0.3)	0.024	10 (0.4)	10 (0.4)	0.000
Dependence on wheelchair	10 (0.3)	10 (0.3)	0.013	10 (0.4)	10 (0.4)	0.000
Medical utilization/procedures, n (%)						
Office or other outpatient services	1893 (63.4)	2043 (54.1)	0.191	1369 (60.6)	1339 (59.3)	0.027
Preventive medicine services	188 (6.3)	308 (8.2)	0.072	167 (7.4)	160 (7.1)	0.012
Emergency department services	98 (3.3)	101 (2.7)	0.036	63 (2.8)	63 (2.8)	0.000
Hospital inpatient and observation care services	40 (1.3)	98 (2.6)	0.090	35 (1.6)	39 (1.7)	0.014
Surgical procedures on the femur and knee joint	13 (0.4)	16 (0.4)	0.002	11 (0.5)	10 (0.4)	0.007
Surgical procedures on the pelvis and hip joint	15 (0.5)	26 (0.7)	0.024	11 (0.5)	14 (0.6)	0.018
Surgical procedures on the spine	12 (0.4)	43 (1.1)	0.084	11 (0.5)	10 (0.4)	0.007
Comorbidities, n (%)						
Disorders of lipoprotein metabolism and other lipidemias	802 (26.9)	1014 (26.8)	0.001	593 (26.3)	617 (27.3)	0.024
Vitamin D deficiency	684 (22.9)	956 (25.3)	0.056	550 (24.4)	545 (24.1)	0.005
Hypertensive diseases	612 (20.5)	823 (21.8)	0.031	457 (20.2)	454 (20.1)	0.003
Chronic lower respiratory diseases	230 (7.7)	365 (9.7)	0.070	177 (7.8)	181 (8.0)	0.007
Other forms of heart disease	198 (6.6)	366 (9.7)	0.112	163 (7.2)	180 (8.0)	0.028
Diabetes mellitus	176 (5.9)	284 (7.5)	0.065	141 (6.2)	134 (5.9)	0.013
Diseases of arteries, arterioles and capillaries	115 (3.9)	213 (5.6)	0.084	99 (4.4)	94 (4.2)	0.011
Ischemic heart diseases	91 (3.0)	192 (5.1)	0.103	76 (3.4)	79 (3.5)	0.007
Overweight and obesity	90 (3.0)	145 (3.8)	0.045	75 (3.3)	66 (2.9)	0.023
Other rheumatoid arthritis	90 (3.0)	163 (4.3)	0.069	75 (3.3)	80 (3.5)	0.012
Anemia, unspecified	85 (2.8)	179 (4.7)	0.099	75 (3.3)	73 (3.2)	0.005
Chronic kidney disease	106 (3.6)	133 (3.5)	0.002	74 (3.3)	79 (3.5)	0.012
Rheumatoid arthritis with rheumatoid factor	71 (2.4)	82 (2.2)	0.014	56 (2.5)	59 (2.6)	0.008
Diseases of liver	67 (2.2)	94 (2.5)	0.016	43 (1.9)	47 (2.1)	0.013
Cerebrovascular diseases	47 (1.6)	101 (2.7)	0.076	40 (1.8)	47 (2.1)	0.023
Heart failure	34 (1.1)	75 (2.0)	0.068	28 (1.2)	33 (1.5)	0.019
Malnutrition	27 (0.9)	46 (1.2)	0.031	23 (1.0)	21 (0.9)	0.009
Systemic lupus erythematosus	22 (0.7)	34 (0.9)	0.018	18 (0.8)	18 (0.8)	0.000
Unspecified dementia	10 (0.3)	10 (0.3)	0.013	10 (0.4)	10 (0.4)	0.000
Ankylosing spondylitis	10 (0.3)	11 (0.3)	0.008	10 (0.4)	10 (0.4)	0.000
Medications, n (%)						
Corticosteroids for systemic use	658 (22.0)	920 (24.4)	0.055	501 (22.2)	501 (22.2)	0.000
HMG CoA reductase inhibitors	471 (15.8)	724 (19.2)	0.089	350 (15.5)	435 (19.3)	0.099
Antidepressants	388 (13.0)	749 (19.8)	0.185	310 (13.7)	396 (17.5)	0.105
NSAIDs	356 (11.9)	662 (17.5)	0.158	294 (13.0)	347 (15.4)	0.067
Opioids	324 (10.9)	598 (15.8)	0.147	280 (12.4)	273 (12.1)	0.009
Antiepileptics	304 (10.2)	625 (16.5)	0.188	241 (10.7)	320 (14.2)	0.106
Diuretics	243 (8.1)	399 (10.6)	0.083	198 (8.8)	209 (9.3)	0.017
Sex hormones and modulators of the genital system	191 (6.4)	373 (9.9)	0.127	166 (7.4)	180 (8.0)	0.023
Alendronate	179 (6.0)	300 (7.9)	0.076	128 (5.7)	187 (8.3)	0.103
Aspirin	113 (3.8)	235 (6.2)	0.112	93 (4.1)	109 (4.8)	0.034
Antipsychotics	97 (3.3)	132 (3.5)	0.014	85 (3.8)	59 (2.6)	0.066
Calcium	84 (2.8)	89 (2.4)	0.029	63 (2.8)	50 (2.2)	0.037
Zoledronic acid	31 (1.0)	26 (0.7)	0.038	27 (1.2)	14 (0.6)	0.061
Raloxifene	27 (0.9)	27 (0.7)	0.021	22 (1.0)	14 (0.6)	0.040
Risedronate	20 (0.7)	43 (1.1)	0.049	18 (0.8)	27 (1.2)	0.040
Ibandronate	19 (0.6)	42 (1.1)	0.051	14 (0.6)	23 (1.0)	0.044
Calcitonin	10 (0.3)	10 (0.3)	0.013	10 (0.4)	10 (0.4)	0.000
Laboratory, n (%)						
Body mass index (BMI), ≧ 30 kg/m^2^	284 (9.5)	395 (10.5)	0.031	221 (9.8)	217 (9.6)	0.006
Calcium in serum, < 6.5 mg/dL	10 (0.3)	10 (0.3)	0.013	10 (0.4)	10 (0.4)	0.000
eGFR, < 60 mL/min/1.73 m^2^	423 (14.2)	443 (11.7)	0.073	294 (13.0)	287 (12.7)	0.009
Phosphate in serum, < 3.5 mg/dL	381 (12.8)	515 (13.6)	0.026	290 (12.8)	292 (12.9)	0.003
Calcidiol in serum, < 30 ng/mL	98 (3.3)	196 (5.2)	0.095	86 (3.8)	82 (3.6)	0.009

PSM: propensity score matching, PTH: Parathyroid hormone, SMD: standardized mean difference, SD: standard deviation, HMG CoA: hydroxy-3-methylglutaryl coenzyme A, NSAIDs: anti-inflammatory and anti-rheumatic products, non-steroids, eGFR: Glomerular filtration rate/1.73 sq M. predicted in serum, plasma or blood by creatinine-based formula

If the patient is less or equal to 10, results show the count as 10

Bold font represents a standardized difference was more than 0.1

^a^ Propensity score matching was performed on age, sex, race, family history of osteoporosis, social economic status, lifestyles, medical utilization/procedures, comorbidities, medication usage (corticosteroids, sex hormones, opioids), and laboratory results (calcium, phosphate, calcidiol, BMI, and eGFR)

###  Fracture, mortality, and calcium abnormalities

**Fig. 2:**
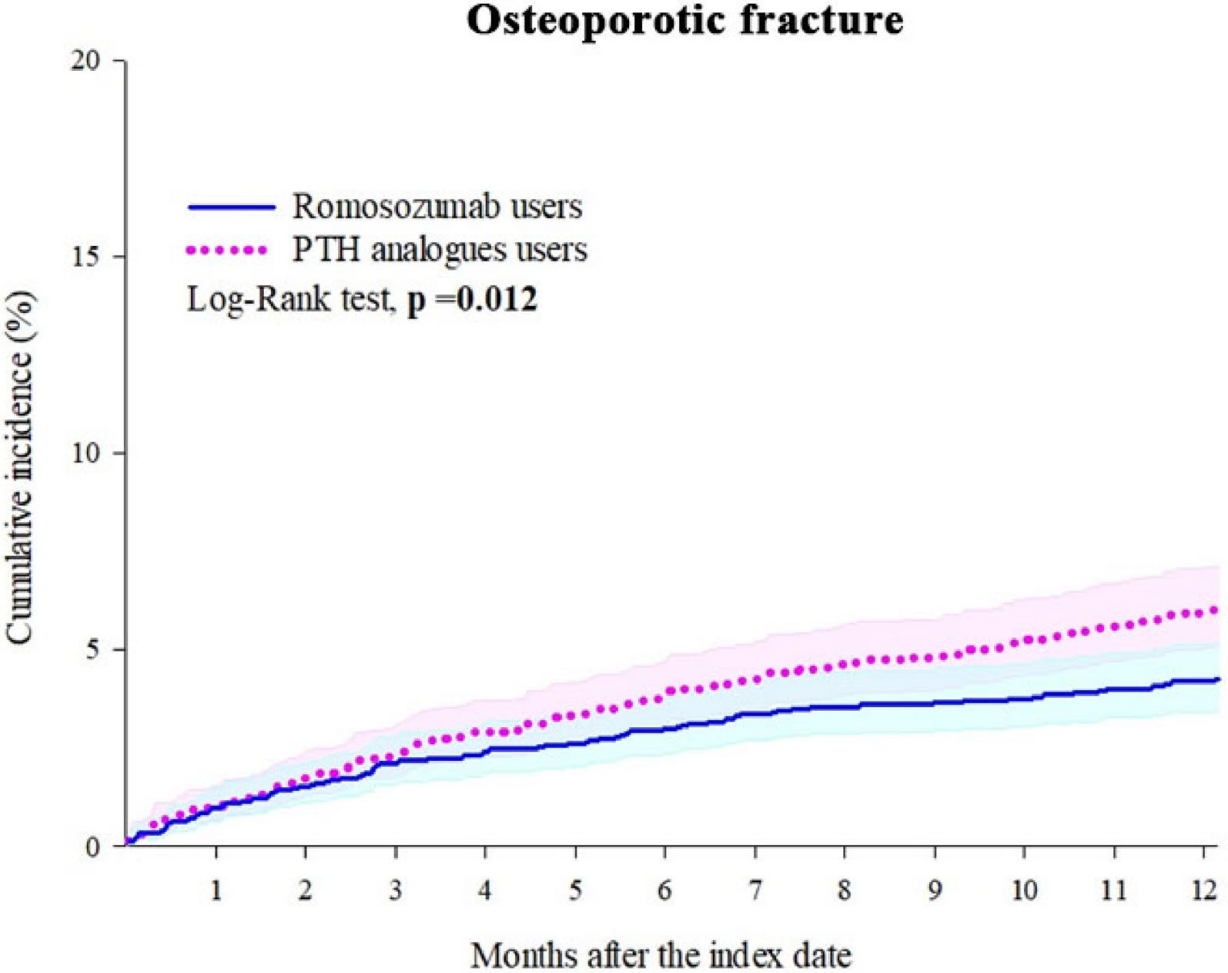
Kaplan–Meier curves for osteoporotic fracture: individuals receiving ≥ 2 doses of romosozumab (ROMO) compared with those receiving ≥ 2 doses of teriparatide (TPTD) or abaloparatide (APTD) during 1-year follow-up

After PSM, ROMO cohort was associated with a significantly lower risk of osteoporotic fractures (HR: 0.711, 95% CI: 0.542–0.931) compared with TPTD/APTD users (Table 2). No significant differences were observed for vertebral fractures (HR: 0.704, 95% CI: 0.458–1.080), non-vertebral fractures (HR: 0.697, 95% CI: 0.425–1.141), hip fractures (HR: 0.365, 95% CI: 0.097–1.376), or all-cause mortality (HR: 0.707, 95% CI: 0.284–1.758), though risks were numerically lower with ROMO users. ROMO was also associated with a lower risk of hypercalcemia (HR: 0.707, 95% CI: 0.511–0.977) and a nonsignificant trend toward higher hypocalcemia (HR: 1.655, 95% CI: 0.892–3.071). Kaplan–Meier analysis demonstrated a significant difference in osteoporotic fractures (log-rank *p* = 0.012; Fig. 2).

**Table 2:** Risk of outcomes from day 1 to 1 year

Outcomes	Patients with outcome		Hazard ratio (95% CI)^a^
	Romosozumab users (n = 2258)	PTH analogs users (n = 2258)	
Osteoporotic fractures	91	125	0.711 (0.542–0.931)*
Vertebral fractures	36	50	0.704 (0.458–1.080)
Non-vertebral fractures	27	38	0.697 (0.425–1.141)
Hip fractures	10	10	0.365 (0.097–1.376)
All-cause mortality	10	11	0.707 (0.284–1.758)
Hypocalcemia	27	16	1.655 (0.892–3.071)
Hypercalcemia	63	87	0.707 (0.511–0.977)*

^*^ p < 0.05

PTH: Parathyroid hormone, CI: Confidence interval. NA: Not available

If the patient is less or equal to 10, results show the count as 10

^a^ Propensity score matching was performed on age, sex, race, family history of osteoporosis, social economic status, lifestyles, medical utilization/procedures, comorbidities, medication usage (corticosteroids, sex hormones, opioids), and laboratory results (calcium, phosphate, calcidiol, BMI, and eGFR)

### Subgroup analysis

**Fig. 3:**
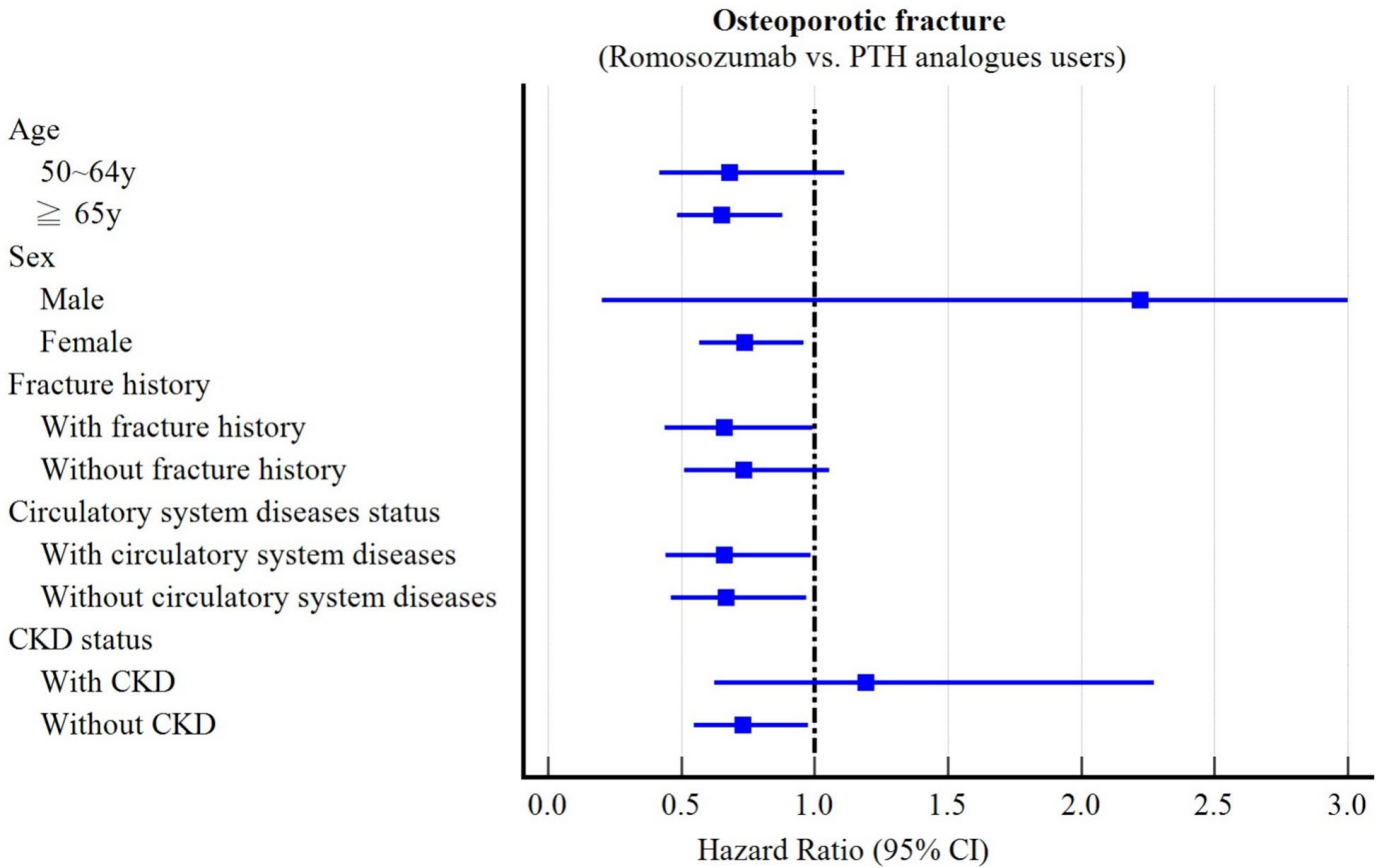
Subgroup analyses: risk of outcomes from day 1 to 1 year stratified by sex, age, fracture history, circulatory system diseases, and chronic renal disease

Sex: Among females (n = 2179 matched pairs), ROMO use was associated with a lower risk of osteoporotic fractures (HR: 0.738, 95% CI: 0.567–0.960) (Supplementary Table 2, Fig. 3). In contrast, no significant difference was observed among males (n = 22 pairs; HR: 2.219, 95% CI: 0.201–24.50), likely reflecting the extremely small size in this subgroup. The formal test for interaction (p interaction = 0.371) was non-significant, suggesting that the treatment effect did not significantly differ between the subgroups. This result, however, should be interpreted with caution, as the small sample size in the male group may have limited the power to detect a true interaction effect. In females, ROMO was also associated with a reduced risk of hypercalcemia (HR: 0.699, 95% CI: 0.501–0.976). No significant differences were observed for vertebral, non-vertebral, or hip fractures, all-cause mortality, or hypocalcemia. Among males, all effect estimates were imprecise due to the extremely small sample size, and no significant associations were detected.

Age: Among patients aged ≥ 65 years (n = 1582 matched pairs), ROMO use was associated with a reduced risk of osteoporotic fractures (HR: 0.652, 95% CI: 0.484–0.879) (Supplementary Table 3). No significant differences were observed in patients aged 50–64 years (n = 732 pairs; HR: 0.681, 95% CI: 0.417–1.112). The interaction test was non-significant (p = 0.881), indicating no statistically significant heterogeneity of treatment by age. In both age groups, no significant differences were found for vertebral fractures, non-vertebral fractures, hip fractures, all-cause mortality, hypocalcemia, or hypercalcemia.

Fracture history: Among patients with prior fractures (n = 250 matched pairs), ROMO use was associated with reduced risks of osteoporotic fractures (HR: 0.659, 95% CI: 0.439–0.991) and vertebral fractures (HR: 0.544, 95% CI: 0.300–0.987) (Supplementary Table 4). In patients without a fracture history (n = 1,944 pairs), no significant differences were observed for osteoporotic fractures (HR: 0.735, 95% CI: 0.511–1.056) or vertebral fractures (HR: 0.848, 95% CI: 0.459–1.567). ROMO as not associated with hypercalcemia in patients with prior fractures (HR: 1.118, 95% CI: 0.431–2.899), whereas a significant association was observed in those without fracture history (HR 0.579, 95% CI 0.399–0.841). All interaction p values were nonsignificant (osteoporotic fracture: p = 0.694; vertebral fracture: p = 0.308; hypercalcemia: p = 0.207) indicating no statistically significant heterogeneity by fracture history. No significant differences were observed for nonvertebral fractures, hip fractures, all-cause mortality, or hypocalcemia in either subgroup.

Circulatory system diseases: In patients with circulatory disease (n = 762 matched pairs), ROMO use was associated with a reduced risk of osteoporotic fractures (HR: 0.660, 95% CI: 0.442–0.986) (Supplementary Table 5). A similar association was observed in those without circulatory disease (n = 1,419 pairs; HR 0.668, 95% CI 0.461–0.968). ROMO use was associated with a reduced risk of vertebral fractures in patients with circulatory disease (HR: 0.448, 95% CI: 0.226–0.887), whereas no significant difference was observed in those without circulatory disease (HR 0.681, 95% CI 0.373–1.242). ROMO was not associated with hypercalcemia in patients with circulatory disease (HR 0.717, 95% CI 0.448–1.149), while a significant association was detected in those without circulatory disease (HR 0.553, 95% CI 0.342–0.894). All interaction p values were nonsignificant (osteoporotic fracture: p = 0.965; vertebral fracture: p = 0.367; hypercalcemia: p = 0.449) indicating no statistically significant heterogeneity by circulatory disease status. In both subgroups, no significant differences were observed for nonvertebral fractures, hip fractures, all-cause mortality, or hypocalcemia.

CKD: Among patients without CKD (n = 1882 matched pairs), ROMO use was associated with reduced risks of osteoporotic fractures (HR: 0.731, 95% CI: 0.547–0.977), non-vertebral fractures (HR: 0.584, 95% CI: 0.344–0.989), and hypercalcemia (HR: 0.599, 95% CI: 0.406–0.883) (Supplementary Table 6). Conversely, among patients with CKD (n = 347 pairs), ROMO use was not associated with significant differences in osteoporotic fractures (HR: 1.191, 95% CI: 0.624–2.273), nonvertebral fractures (HR: 1.403, 95% CI: 0.445–4.421), or hypercalcemia (HR: 0.775, 95% CI: 0.385–1.558). All interaction p values were nonsignificant (osteoporotic fracture: p = 0.176; nonvertebral fracture: p = 0.173; hypercalcemia: p = 0.527), indicating no statistically significant heterogeneity by CKD status and likely reflecting limited power due to the relatively small CKD sample. No significant differences were found for vertebral fractures, hip fractures, all-cause mortality, or hypocalcemia in either subgroup.

### Sensitivity analyses

First, after adjusting for an alternative set of covariates, the results remained consistent (Supplementary Table 7). Second, using the TriNetX Global Network to assess geographic differences, ROMO users showed nonsignificant trends toward lower risks of osteoporotic fractures (HR: 0.823, 95% CI: 0.637–1.064), vertebral fractures (HR: 0.731, 95% CI: 0.472–1.133), non-vertebral fractures (HR: 0.850, 95% CI: 0.548–1.319), hip fractures (HR: 0.489, 95% CI: 0.147–1.624), and all-cause mortality (HR: 0.891, 95% CI: 0.378–2.098) (Supplementary Table 8). A significant reduction in hypercalcemia risk was observed (HR: 0.639, 95% CI: 0.459–0.889), while no significant difference was found in the risk of hypocalcemia (HR: 1.565, 95% CI: 0.853–2.872).

Third, in a head-to-head comparison with TPTD users, ROMO cohort showed a significantly reduced risk of non-vertebral fractures (HR: 0.509, 95% CI: 0.279–0.931) and hypercalcemia (HR: 0.645, 95% CI: 0.439–0.947) (Table 3), while no significant differences were observed for other outcomes. Due to a small sample size (n < 10), hip fracture and mortality analyses were not available. Kaplan–Meier analysis indicated a nonsignificant trend toward a lower risk of osteoporotic fractures in ROMO users compared to TPTD users (log-rank p = 0.105; Supplementary Fig. 1).

**Table 3:** Risk of outcomes from day 1 to 1 year in a modified control cohort restricted to teriparatide users only

Outcomes	Patients with outcome		Hazard ratio (95% CI)^a^
	Romosozumab users (n = 1586)	Teriparatide users (n = 1586)	
Osteoporotic fractures	75	95	0.779 (0.576–1.055)
Vertebral fractures	32	42	0.753 (0.475–1.192)
Non-vertebral fractures	16	31	0.509 (0.279–0.931)*
Hip fractures	< 10	< 10	NA
All-cause mortality	< 10	< 10	NA
Hypocalcemia	20	12	1.652 (0.808–3.380)
Hypercalcemia	43	66	0.645 (0.439–0.947)*

^*^ p < 0.05

CI: Confidence interval. NA: Not available

^a^ Propensity score matching was performed on age, sex, race, family history of osteoporosis, social economic status, lifestyles, medical utilization/procedures, comorbidities, medication usage (corticosteroids, sex hormones, opioids), and laboratory results (calcium, phosphate, calcidiol, BMI, and eGFR)

Fourth, we extended the observation window to 366–730 days (i.e., the second treatment year) to examine the robustness of our findings beyond the initial 12-month period. Compared with presumed TPTD-continued patients, presumed ROMO-off patients exhibited a lower risk of hypercalcemia (HR: 0.487, 95% CI: 0.304–0.780) and nonsignificant trends toward reduced risks of osteoporotic fractures (HR: 0.739, 95% CI: 0.522–1.046), vertebral fractures (HR: 0.641, 95% CI: 0.348–1.182), and non-vertebral fractures (HR: 0.618, 95% CI: 0.337–1.133) (Supplementary Table 9). Data on hip fractures, mortality, and hypocalcemia were not reported due to small sample sizes.

## Discussion

Osteoporosis reflects accelerated but imbalanced remodeling, resulting in cancellous and cortical bone loss with microarchitectural deterioration [1]. Restoring balance is the therapeutic goal, with signaling pathways central to treatment [32, 33]. Antiresorptive agents slow bone loss but cannot fully restore balance and may cause rare long-term complications such as osteonecrosis of the jaw or atypical femoral fracture [34]. For patients requiring potent anabolic effects without suppression-related adverse effects, TPTD is often chosen [4].

Recent studies suggest ROMO provides greater BMD gains, favorable safety, and higher adherence than TPTD [21, 35]. A network meta-analysis found that TPTD, APTD, and ROMO all reduced vertebral, non-vertebral, and hip fractures versus placebo, without significant inter-drug differences [36]. In our TriNetX U.S. analysis, after propensity matching, ROMO offered stronger protection against osteoporotic fractures, particularly in older (≥ 65 years old) women with prior fractures (3 months–3 years), with or without circulatory disease, or without CKD. ROMO reduced vertebral fracture risk in patients with fracture history or circulatory disease and non-vertebral fracture risk in those without CKD. These effects, consistent with rapid and greater BMD gains, were most evident during the first 12 months [21]. However, small sample sizes and missing subgroup data limit generalization, especially to men.

Subgroup analyses suggested hypercalcemia was less frequent with ROMO, especially in women without fracture history, circulatory disease, or CKD. Hypocalcemia occurred more often, particularly in women aged ≥ 65 years, without fracture history, and with benefits consistent across circulatory diseases status and CKD, although not statistically significant, underscoring the need for monitoring. Head-to-head comparison also showed ROMO carried a lower hypercalcemia risk without significantly increasing hypocalcemia versus TPTD.

Both APTD and TPTD increased BMD at the total hip, femoral neck, and lumbar spine at 6–18 months versus placebo [16]. APTD exceeded TPTD at most sites except the lumbar spine at 18 months, with significantly reduced major osteoporotic fracture risk. Bone turnover markers [procollagen type I N-terminal propeptide (PINP) and carboxy-terminal cross-linking telopeptide of type I collagen (CTX)] rose more with TPTD, indicating greater remodeling activity, although differences were nonsignificant [16]. Because efficacy differences were modest [3, 16], APTD was excluded from the main comparison. Compared with TPTD, ROMO demonstrated significant reductions in non-vertebral fractures and hypercalcemia, with residual benefit of hypercalcemia persisting after discontinuation, largely reflecting higher TPTD risk.

All three agents are generally safe but carry agent-specific warnings. The boxed 24-month limit for TPTD related to osteosarcoma risk was removed after 18 years of surveillance [10, 11], although it remains in place for APTD. ROMO is restricted to 12 months and carries an FDA warning against use in patients with recent CV events [18, 37], although the evidence underlying this concern remains debated [38, 39]. CV risk signals for ROMO are inconsistent: CV events were comparable between ROMO and placebo in postmenopausal women, and no excess risk over bisphosphonates has been demonstrated [19, 40]; however, several findings raise caution. In ARCH and a phase III trial in men, adjudicated serious CV events occurred more often with ROMO (2.5% vs. 1.9% vs. alendronate; 4.9% vs. 2.5% vs. placebo) [18, 37]. Pharmacovigilance data from Japan also suggest potential excess risk [41, 42], and multivariate analyses show higher odds of cardiac events in patients with pre-existing cardiac disease (OR = 5.9) or hypertension (OR = 1.6), and higher cerebrovascular risk in those with cerebrovascular disease (OR = 2.7) or hypertension (OR = 2.6) [42]. A meta-analysis of randomized trials further indicated that ROMO may increase composite arterial events compared with placebo, whereas abaloparatide was associated with lower odds of major adverse CV events or death (MACE) (OR = 0.25–0.31); these associations were attenuated when PTH analogues were pooled [43].

In contrast, a recent TriNetX analysis reported lower 1-year 3-point MACE rates with ROMO than with PTH analogues (158 vs. 211; P = 0.003), including fewer myocardial ischemic events (31 vs. 58; P = 0.003) and fewer cerebrovascular events (56 vs. 79; P = 0.037), along with a nonsignificant trend toward lower mortality [35]. These real-world findings suggest ROMO may not confer higher short-term CV risk, but the overall evidence remains conflicting and will require larger, more diverse, and methodologically rigorous studies to clarify.

In treatment-naïve patients or those previously exposed to antiresorptive agents, ROMO appeared more protective, likely reflecting its antiresorptive effect compared with the resorption increase seen with TPTD/APTD and TPTD alone. However, given the modest differences in efficacy, the extent to which ROMO is truly superior for individual patients remains uncertain [38, 39]. Beyond 12-month ROMO treatment window, discontinuation or transition to antiresorptive therapy has shown nonsignificant trends toward fewer osteoporotic, vertebral, and non-vertebral fractures compared with continued TPTD; nevertheless, prolonged antiresorptive therapy without intermittent remodeling raises concerns regarding microdamage accumulation.

Sequential strategies designed to maintain or further enhance prior BMD gains generally outperform prolonged monotherapy, with the important exception of transitions after denosumab, where rebound bone loss complicates subsequent management [30, 44–47]. Prior antiresorptive therapy may attenuate anabolic responses [20, 21], including the otherwise greater BMD gains achieved with ROMO compared with TPTD [27]. Post-anabolic antiresorptive consolidation or cyclic anabolic-antiresorptive regimens, therefore, remain essential in patients at very high fracture risk. Notably, sustained antifracture benefits for up to 30 months have been reported with TPTD followed by antiresorptive therapy [48], suggesting that TPTD may be preferable for patients with lower hypercalcemia risk who are likely require longer-term sequential or cyclic treatment approaches.

### Limitations

This study has several limitations. First, reliance on the TriNetX U.S. network may limit generalizability, although findings were consistent in the Global network. Second, TriNetX is not a population-based dataset; its data are derived solely from participating HCOs. Medical encounters occurring outside the network are therefore not captured, which may result in incomplete follow-up and potential misclassification of exposures or outcomes when key clinical events take place elsewhere. Because TriNetX reflects only the population served by contributing HCOs, selection bias is possible, and the findings may not be fully generalizable to broader populations. Third, residual confounding and misclassification are inherent to electronic health records—based studies. Differences between TPTD and APTD may have introduced bias, although this was partially mitigated by a sensitivity analysis comparing ROMO with TPTD. Injection frequency and treatment duration differed across patients, which may have affected adherence. Baseline T-scores, trabecular bone scores, bone turnover markers, and supplementation data (e.g., vitamin D and calcium) were also incomplete. To reduce misclassification, we included patients with ≥ 2 osteoporosis diagnoses and no recent fractures. Finally, the applicability of these findings to non-Western populations remains uncertain.

## Conclusions

In this real-world analysis, ROMO provided greater fracture protection than TPTD/APTD during the first year, with consistent trends in vertebral, non-vertebral, and hip fractures. Benefits were most evident in women with prior fractures or without CKD, regardless of circulatory disease status. ROMO showed a lower risk of hypercalcemia but a nonsignificant trend toward hypocalcemia, highlighting the need for monitoring. Despite small, matched samples, dosing differences, and limited follow-up, these findings support ROMO as a valuable option for severe osteoporotic women requiring rapid, short-term protection, especially after BPs. Therapy should remain individualized, considering fracture risk, comorbidities, safety, and long-term management strategies.

## Supplementary Information

Supplementary material 1.

## Data Availability

The corresponding author will on request detail the restrictions and any conditions under which access to some data may be provided.
