# Supplementary material for: Real-world evidence indicates romosozumab use is associated with a greater reduction in osteoporotic fractures than PTH (1–34) analogs in women
Source: Biol Sex Differ. 2026 Jan 3;17:21. doi: 10.1186/s13293-025-00817-1 (PMC12870266; doi:10.1186/s13293-025-00817-1)
Supplement: Supplementary file 1 — Supplementary material 1. [file 13293_2025_817_MOESM1_ESM.pdf]

Supplementary Table 1 Codes for baseline of characteristics of study subjects

Variables	Codes
**Demographic covariates**	
Social economic status (SES, persons with potential health hazards related to socioeconomic and psychosocial circumstances)	ICD-10-CM: Z55-Z65
Family history of osteoporosis	ICD-10-CM: Z82.62
**Lifestyle-related variables**	
Nicotine dependence	ICD-10-CM: F17
Personal history of nicotine dependence	ICD-10-CM: Z87.891
Tobacco use	ICD-10-CM: Z72.0
Alcohol related disorders	ICD-10-CM: F10
Reduced mobility	ICD-10-CM: Z74.0
Difficulty in walking, not elsewhere classified	ICD-10-CM: R26.2
Dependence on wheelchair	ICD-10-CM: Z99.3
**Medical utilization/procedures**	
Office or other outpatient services	CPT: 1013626
Emergency department services	CPT: 1013711
Hospital inpatient services	CPT: 1013659
Preventive medicine services	CPT: 1013829
Surgical procedures on the femur and knee joint	CPT: 1004987
Surgical procedures on the pelvis and hip joint	CPT: 1004841
Surgical procedures on the spine	CPT: 1004038
**Comorbidities**	
Disorders of lipoprotein metabolism and other lipidemias	ICD-10-CM: E78
Vitamin D deficiency	ICD-10-CM: E55
Hypertensive diseases	ICD-10-CM: I10-I1A
Chronic lower respiratory diseases	ICD-10-CM: J40-J4A
Other forms of heart disease	ICD-10-CM: I30-I5A
Diabetes mellitus	ICD-10-CM: E08-E13
Diseases of arteries, arterioles and capillaries	ICD-10-CM: I70-I79
Ischemic heart diseases	ICD-10-CM: I20-I25
Overweight and obesity	ICD-10-CM: E66
Other rheumatoid arthritis	ICD-10-CM: M06
Anemia, unspecified	ICD-10-CM: D64.9
Chronic kidney disease (CKD)	ICD-10-CM: N18
Rheumatoid arthritis with rheumatoid factor	ICD-10-CM: M05

Diseases of liver	ICD-10-CM: K70-K77
Cerebrovascular diseases	ICD-10-CM: I60-I69
Heart failure	ICD-10-CM: I50
Malnutrition	ICD-10-CM: E40-E46
Systemic lupus erythematosus (SLE)	ICD-10-CM: M32
Unspecified dementia	ICD-10-CM: F03
Ankylosing spondylitis	ICD-10-CM: M45
**Medication usage**	
Corticosteroids for systemic use	ATC: H02
HMG CoA reductase inhibitors	ATC: C10AA
Antidepressants	ATC: N06A
NSAIDs	ATC: M01A
Opioids	ATC: N02A
Antiepileptics	ATC: N03
Diuretics	ATC: C03
Sex hormones and modulators of the genital system	ATC: G03
Alendronate	ATC: M05BA04
Aspirin	ATC: N02BA01
Antipsychotics	ATC: N05A
Calcium	ATC: A12AA
Zoledronic acid	ATC: M05BA08
Raloxifene	ATC: G03XC01
Risedronate	ATC: M05BA07
Ibandronate	ATC: M05BA06
Calcitonin	ATC: H05BA
**Laboratory measurements**	
Body mass index	TNX curated: 9083
Calcium in serum	TNX curated: 9022
eGFR	TNX curated: 8001
Phosphate in serum	TNX curated: 9027
Calcidiol in serum	TNX curated: 9034

Note:
ICD-10-CM: International Classification of Diseases, Tenth Revision, Clinical Modification; CPT: Current Procedural Terminology; ATC: Anatomical Therapeutic Chemical Classification System; TNX: TriNetX; HMG CoA: hydroxy-3-methylglutaryl coenzyme A; NSAIDs: anti-inflammatory and anti-rheumatic products, non-steroids; eGFR: Glomerular filtration rate/1.73 m^2 predicted in serum, plasma by creatinine-based formula.

Supplementary Table 2 Risk of outcomes from day 1 to 1 year stratified by sex

Outcomes (Romosozumab vs. PTH analog users)	Hazard ratio (95% CI)[a]		p for interaction
	Male (n = 22 pairs)	Female (n = 2179 pairs)	
Osteoporotic fractures	2.219 (0.201-24.50)	0.738 (0.567-0.960)*	0.371
Vertebral fractures	1.106 (0.069-17.67)	0.717 (0.464-1.108)	0.762
Non-vertebral fractures	NA	0.752 (0.475-1.191)	NA
Hip fractures	NA	0.356 (0.113-1.118)	NA
All-cause mortality	NA	0.760 (0.283-2.042)	NA
Hypocalcemia	NA	1.666 (0.898-3.093)	NA
Hypercalcemia	NA	0.699 (0.501-0.976)*	NA

Note:
PTH: Parathyroid hormone, CI: Confidence interval. NA: Not available.
[a.] Propensity score matching was performed on age, sex, race, family history of osteoporosis, social economic status, lifestyles, medical utilization/procedures, comorbidities, medication usage (corticosteroids, sex hormones, opioids), and laboratory results (calcium, phosphate, calcidiol, BMI, and eGFR).
* $p < 0.05$.

Supplementary Table 3 Risk of outcomes from day 1 to 1 year stratified by age

Outcomes (Romosozumab vs. PTH analog users)	Hazard ratio (95% CI)[a]		p for interaction
	50~64 years old (n = 732 pairs)	≥65 years old (n = 1582 pairs)	
Osteoporotic fractures	0.681 (0.417-1.112)	0.652 (0.484-0.879)*	0.881
Vertebral fractures	0.589 (0.258-1.346)	0.634 (0.400-1.005)	0.878
Non-vertebral fractures	0.690 (0.263-1.814)	0.610 (0.349-1.067)	0.828
Hip fractures	NA	0.365 (0.097-1.375)	NA
All-cause mortality	0.974 (0.061-15.57)	1.293 (0.449-3.727)	0.851
Hypocalcemia	1.143 (0.384-3.401)	1.960 (0.951-4.042)	0.419
Hypercalcemia	0.552 (0.280-1.090)	0.848 (0.583-1.233)	0.278

Note:
PTH: Parathyroid hormone, CI: Confidence interval. NA: Not available.
[a.] Propensity score matching was performed on age, sex, race, family history of osteoporosis, social economic status, lifestyles, medical utilization/procedures, comorbidities, medication usage (corticosteroids, sex hormones, opioids), and laboratory results (calcium, phosphate, calcidiol, BMI, and eGFR).
* p <0.05.

Supplementary Table 4 Risk of outcomes from day 1 to 1 year stratified by fracture history

Outcomes (Romosozumab vs. PTH analog users)	Hazard ratio (95% CI)[a]		p for interaction
	With fracture history[b] (n = 250 pairs)	Without fracture history[c] (n = 1944 pairs)	
Osteoporotic fractures	0.659 (0.439-0.991)*	0.735 (0.511-1.056)	0.694
Vertebral fractures	0.544 (0.300-0.987)*	0.848 (0.459-1.567)	0.308
Non-vertebral fractures	1.004 (0.451-2.236)	0.667 (0.360-1.236)	0.427
Hip fractures	1.509 (0.252-9.029)	NA	NA
All-cause mortality	0.666 (0.111-3.989)	1.116 (0.405-3.077)	0.622
Hypocalcemia	NA	1.615 (0.831-3.138)	NA
Hypercalcemia	1.118 (0.431-2.899)	0.579 (0.399-0.841)*	0.207

Note:
PTH: Parathyroid hormone, CI: Confidence interval. NA: Not available.
[a] Propensity score matching was performed on age, sex, race, family history of osteoporosis, social economic status, lifestyles, medical utilization/procedures, comorbidities, medication usage (corticosteroids, sex hormones, opioids), and laboratory results (calcium, phosphate, calcidiol, BMI, and eGFR).
[b] Fractures occurred within 3 years on or before the index date.
[c] Fractures never occurred within 3 years on or before the index date.

Supplementary Table 5 Risk of outcomes from day 1 to 1 year stratified by circulatory system diseases

Outcomes (Romosozumab vs. PTH analog users)	Hazard ratio (95% CI) [a]		p for interaction
	With circulatory system diseases[b] (n = 762 pairs)	Without circulatory system diseases[c] (n = 1419 pairs)	
Osteoporotic fractures	0.660 (0.442-0.986)*	0.668 (0.461-0.968)*	0.965
Vertebral fractures	0.448 (0.226-0.887)*	0.681 (0.373-1.242)	0.367
Non-vertebral fractures	0.711 (0.349-1.452)	0.615 (0.323-1.172)	0.767
Hip fractures	0.243 (0.027-2.178)	0.737 (0.165-3.292)	0.413
All-cause mortality	1.605 (0.384-6.716)	0.588 (0.140-2.459)	0.331
Hypocalcemia	1.780 (0.658-4.812)	1.486 (0.607-3.635)	0.791
Hypercalcemia	0.717 (0.448-1.149)	0.553 (0.342-0.894)*	0.449

Note:
PTH: Parathyroid hormone, CI: Confidence interval. NA: Not available.
[a] Propensity score matching was performed on age, sex, race, family history of osteoporosis, social economic status, lifestyles, medical utilization/procedures, comorbidities, medication usage (corticosteroids, sex hormones, opioids), and laboratory results (calcium, phosphate, calcidiol, BMI, and eGFR).
[b] Fractures occurred within 3 years on or before the index date.
[c] Fractures never occurred within 3 years on or before the index date.
* $p < 0.05$.

Supplementary Table 6 Risk of outcomes from day 1 to 1 year stratified by chronic kidney disease

Outcomes<br>(Romosozumab vs. PTH analog users)	Hazard ratio (95% CI) [a]		p for interaction
	With CKD[b]<br>(n = 347 pairs)	Without CKD[c]<br>(n = 1882 pairs)	
Osteoporotic fractures	1.191 (0.624-2.273)	0.731 (0.547-0.977)*	0.176
Vertebral fractures	1.405 (0.446-4.427)	0.800 (0.498-1.286)	0.374
Non-vertebral fractures	1.403 (0.445-4.421)	0.584 (0.344-0.989)*	0.173
Hip fractures	NA	0.392 (0.123-1.249)	NA
All-cause mortality	3.981 (0.445-35.61)	0.559 (0.164-1.909)	0.125
Hypocalcemia	6.050 (0.728-50.25)	1.794 (0.860-3.745)	0.287
Hypercalcemia	0.775 (0.385-1.558)	0.599 (0.406-0.883)*	0.527

Note:
PTH: Parathyroid hormone, CI: Confidence interval. NA: Not available.
[a] Propensity score matching was performed on age, sex, race, family history of osteoporosis, social economic status, lifestyles, medical utilization/procedures, comorbidities, medication usage (corticosteroids, sex hormones, opioids), and laboratory results (calcium, phosphate, calcidiol, BMI, and eGFR).
[b] Diagnosed chronic kidney disease (ICD-10-CM code N18) or eGFR < 60 ml/min/1.73m^2 within 1 year on or before the index date.
[c] Never diagnosed chronic kidney disease (ICD-10-CM code N18) or eGFR < 60 ml/min/1.73m^2 within 1 year on or before the index date.
* $p < 0.05$.

Supplementary Table 7 Risk of outcomes from day 1 to 1 year after adjustment for different covariates

Outcomes (Romosozumab vs. PTH analogues users)	Hazard ratio (95% CI)			
	Model 1[a]	Model 2[b]	Model 3[c]	Model 4[d]
Osteoporotic fractures	0.625 (0.502-0.776)*	0.640 (0.497-0.825)*	0.655 (0.505-0.849)*	0.711 (0.542-0.931)*
Vertebral fractures	0.618 (0.435-0.880)*	0.608 (0.401-0.922)*	0.649 (0.423-0.994)*	0.704 (0.458-1.080)
Non-vertebral fractures	0.605 (0.413-0.885)*	0.612 (0.396-0.944)*	0.671 (0.428-1.051)	0.697 (0.425-1.141)
Hip fractures	0.436 (0.173-1.098)	0.406 (0.143-1.153)	0.356 (0.113-1.117)	0.365 (0.097-1.376)
All-cause mortality	0.654 (0.317-1.349)	0.710 (0.285-1.764)	0.527 (0.210-1.321)	0.707 (0.284-1.758)
Hypocalcemia	1.482 (0.892-2.461)	1.836 (0.980-3.437)	1.533 (0.848-2.771)	1.655 (0.892-3.071)
Hypercalcemia	0.708 (0.536-0.934)*	0.678 (0.494-0.930)*	0.694 (0.505-0.954)*	0.707 (0.511-0.977)*

Note:
PTH: Parathyroid hormone, CI: Confidence interval. NA: Not available.
[a] Crude, before matching.
[b] Propensity score matching was performed on age at index, current age, sex, and race.
[c] Propensity score matching was performed on age, sex, race, family history of osteoporosis, social economic status, lifestyles, medical utilization/procedures, and BMI.
[d] Propensity score matching was performed on age, sex, race, family history of osteoporosis, social economic status, lifestyles, medical utilization/procedures, comorbidities, medication usage (corticosteroids, sex hormones, opioids), and laboratory results (calcium, phosphate, calcidiol, BMI, and eGFR).
* $p < 0.05$.

Supplementary Table 8 Risk of outcomes from day 1 to 1 year using the Global Collaborative Network

Outcomes	Patients with outcome		Hazard ratio (95% CI)[a]
	Romosozumab users (n = 2401)	PTH analogues users (n = 2401)	
Osteoporotic fractures	107	128	0.823 (0.637-1.064)
Vertebral fractures	35	47	0.731 (0.472-1.133)
Non-vertebral fractures	37	43	0.850 (0.548-1.319)
Hip fractures	10	10	0.489 (0.147-1.624)
All-cause mortality	10	11	0.891 (0.378-2.098)
Hypocalcemia	27	17	1.565 (0.853-2.872)
Hypercalcemia	58	89	0.639 (0.459-0.889)*

Note:
PTH: Parathyroid hormone, CI: Confidence interval. NA: Not available.
If the patient is less or equal to 10, results show the count as 10.
[a.] Propensity score matching was performed on age, sex, race, family history of osteoporosis, social economic status, lifestyles, medical utilization/procedures, comorbidities, medication usage (corticosteroids, sex hormones, opioids), and laboratory results (calcium, phosphate, calcidiol, BMI, and eGFR).
* $p < 0.05$.
The data for this study were sourced from the TriNetX network, which covers over 120 healthcare organizations across 19 countries, with all data being de-identified and anonymized, and processed in strict compliance with regulatory standards such as HIPAA and GDPR to ensure privacy and data quality.

Supplementary Table 9 Risk of outcomes during extended follow-up (366 to 730 days)

Outcomes	Patients with outcome		Hazard ratio (95% CI)[a]
	Romosozumab users (n = 1586)	Teriparatide users (n = 1586)	
Osteoporotic fractures	56	74	0.739 (0.522-1.046)
Vertebral fractures	17	26	0.641 (0.348-1.182)
Non-vertebral fractures	17	27	0.618 (0.337-1.133)
Hip fractures	<10	<10	NA
All-cause mortality	<10	<10	NA
Hypocalcemia	<10	<10	NA
Hypercalcemia	26	52	0.487 (0.304-0.780)*

Note:
CI: Confidence interval. NA: Not available.
[a] Propensity score matching was performed on age, sex, race, family history of osteoporosis, social economic status, lifestyles, medical utilization/procedures, comorbidities, medication usage (corticosteroids, sex hormones, opioids), and laboratory results (calcium, phosphate, calcidiol, BMI, and eGFR).
* $p < 0.05$.

Supplementary Figure 1

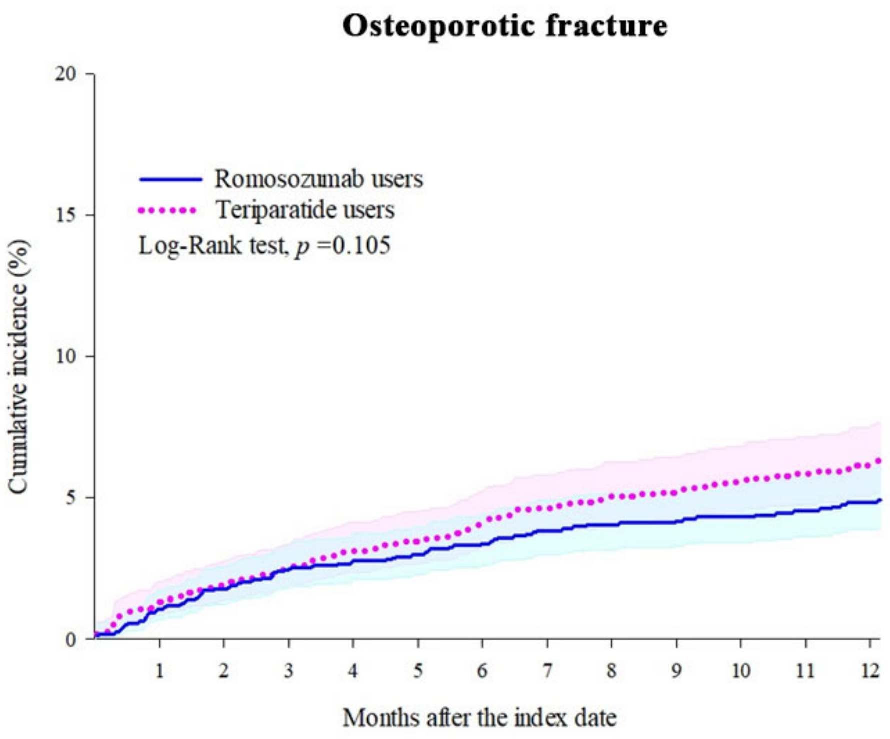

Supplementary Fig. 1 Kaplan-Meier curves for osteoporotic fracture: individuals receiving ≥2 doses of romosozumab (ROMO) compared with those receiving ≥2 doses of teriparatide (TPTD) during 1-year follow-up (extended follow-up from 366 to 730 days after the index date).
